# Sequence space exploration in SHP2: mechanistic insights from disease-associated mutations

**DOI:** 10.1042/BCJ20250266

**Published:** 2026-10-26

**Authors:** Anne E. van Vlimmeren, Ziyuan Jiang, Devon A. Semoy, Neel H. Shah

**Affiliations:** 1 Department of Chemistry, Columbia University, New York, NY 10027, U.S.A.; 2 Department of Biological Sciences, Columbia University, New York, NY 10027, U.S.A.; 3 Herbert Irving Comprehensive Cancer Center, Columbia University, New York, NY 10032, U.S.A.

**Keywords:** cancer, cell signaling, Noonan Syndrome, PTPN11, RASopathy, tyrosine phosphatase

## Abstract

Multi-domain signaling proteins are regulatable switches that mediate signal transduction with high fidelity and selectivity in response to diverse cues. These proteins often harbor disease-associated mutations that disrupt their regulatory properties or interaction specificity to drive pathogenic signaling. Mapping sequence–structure–function relationships in multi-domain signaling proteins is critical to understanding signaling pathways in both healthy and disease states. The protein tyrosine phosphatase SHP2 is a multi-domain protein that can serve as a model system to dissect the molecular basis of signal transduction: SHP2 combines catalytic activity with several binding sites and domains, integrates a variety of inputs, yields a diverse array of potential downstream outcomes, and has extensive interdomain interactions that regulate both its catalytic activity and scaffolding properties. Moreover, numerous mutations have been identified in SHP2 in a variety of human diseases. In the present review, we discuss the myriad ways in which disease-associated mutations perturb SHP2 structure and function. An emerging theme is that pathogenic signaling and disease type are not exclusively determined by mutation-dependent changes in catalytic activity. Rather, SHP2 pathogenicity is driven by changes to its conformational ensemble, which can perturb both catalytic activity and protein–protein interactions. However, these two functional attributes are sometimes differentially affected by mutations, which gives rise to a large diversity of pathogenic outcomes. At the end of the present review, we discuss several open questions and future research directions for understanding SHP2 and multi-domain signaling proteins more broadly.

## Introduction

A large swath of eukaryotic cell signaling is orchestrated by multi-domain proteins that undergo ligand-dependent conformational rearrangements to convert an array of input signals into diverse cellular responses [1–8]. Modular binding domains in these signaling enzymes, such as SH3, SH2, PDZ, and FERM domains^i^, are often multifunctional. They mediate the selective recognition of other biomolecules (e.g. proteins and lipids), thereby controlling protein localization in response to specific upstream signals (inputs), and they regulate downstream protein function (outputs) through intramolecular interactions, often in a manner that depends on the input signal. In other words, modular binding domains allow multi-domain signaling proteins to be responsive switches that are selective and regulatable. In order to coordinate specific input–output relationships in a sea of biomolecules, multi-domain signaling proteins must recognize subtle differences in their binding partners. In order to be regulatable, these proteins must be able to undergo facile conformational changes in response to inputs, and thus, their individual conformational states are relatively metastable. A direct consequence of their marginal selectivity and conformational metastability is that multi-domain signaling proteins are highly susceptible to dysregulation by mutations, which can remodel an interaction interface or perturb a conformational landscape. Understanding the interplay between the biochemical activities of each domain in a multi-domain protein, and how this interplay is perturbed by mutations that lead to aberrant activity, is at the heart of much of cell signaling research.

The non-receptor protein tyrosine phosphatase (PTP) SHP2^ii^, encoded by the gene *PTPN11*, is a quintessential example of a disease-relevant multi-domain protein that can serve as a useful model for dissecting sequence–structure–function relationships in cell signaling. SHP2 is composed of tandem Src-homology 2 (SH2) domains, a catalytic PTP domain, and a disordered C-terminal tail with a proline-rich region and multiple tyrosine phosphorylation sites (Figure 1A) [9,10]. In the basal state, the N-terminal SH2 (N-SH2) domain is docked onto the PTP domain and sterically occludes the active site, with the C-terminal SH2 (C-SH2) domain buttressing this auto-inhibited, or closed, conformation (Figure 1B) [9]. This equilibrium can be shifted toward an active and open state upon binding of tyrosine-phosphorylated ligands to the SH2 domains, which simultaneously relieves auto-inhibition and recruits SHP2 to its signaling partners, directly connecting localization and enzyme activation (Figure 1B,C). In recent years, it has become clear that SHP2 has at least two active states (Figure 1B) [10–14], although the functional differences between these active states are not yet known. The C-terminal tail, which has not been resolved in any experimental structures, fine-tunes SHP2 function by providing additional binding and regulatory sites [15,16]. Through this modular architecture, SHP2 integrates cues from receptor tyrosine kinases, cytokine-, and immune receptors, and scaffolding proteins to control multiple important signaling pathways, including the Ras/MAPK, PI3K/AKT, and JAK/STAT pathways (Figure 1C) [17–23]. These attributes make SHP2 a valuable model to study how a single protein can coordinate a range of different signaling processes.

**Figure 1:**
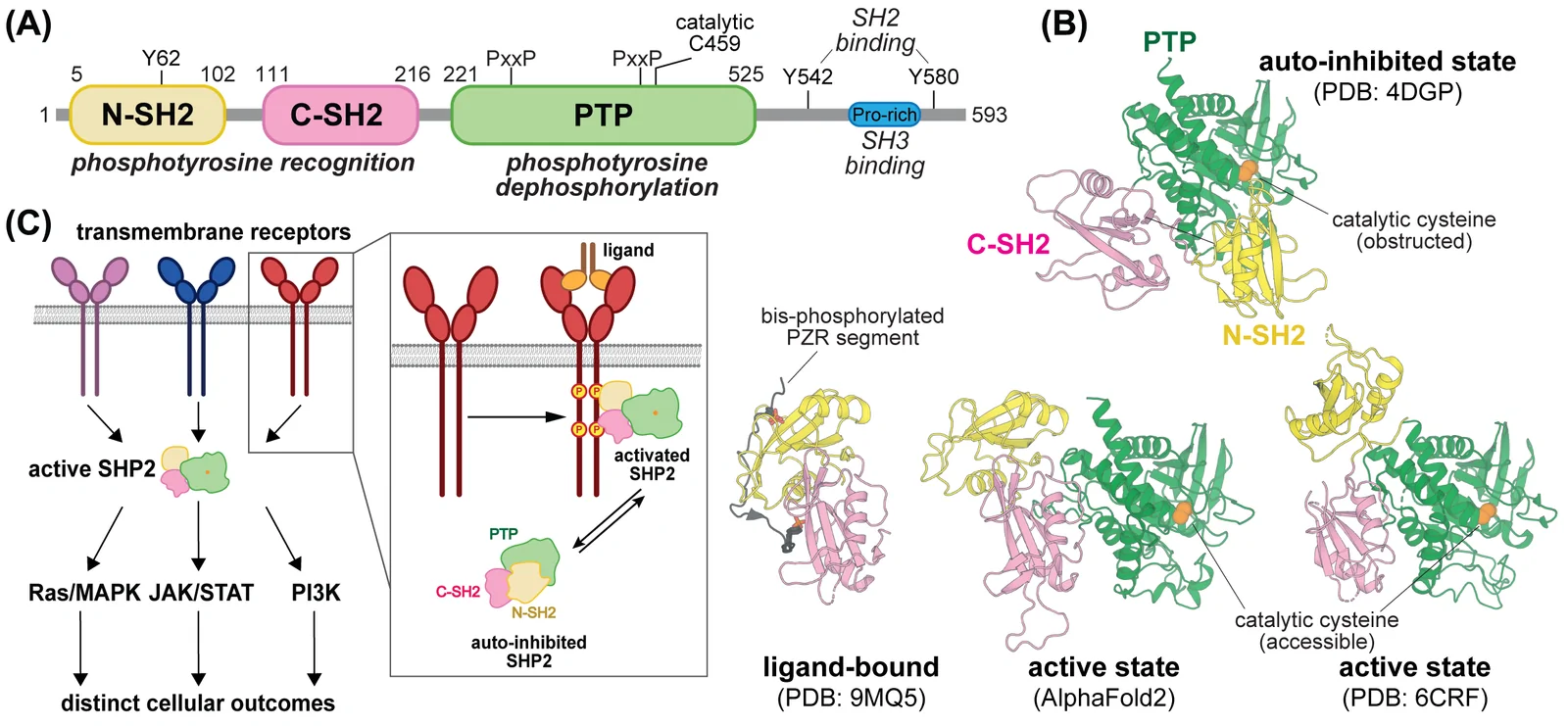
Structure, regulation, and function of the protein tyrosine phosphatase SHP2 (**A**) Domain architecture of SHP2, showing two SH2 domains, a catalytic PTP domain, and a disordered C-terminal tail. Select functionally important sites are highlighted, including prevalent phosphorylated tyrosine residues, various SH3 binding motifs, and the catalytic cysteine. (**B**) Representative experimentally determined and computationally predicted structures of SHP2. *Top:* The auto-inhibited state of wild-type SHP2 (PDB: 4DGP). *Bottom left:* the tandem SHP2 SH2 domains bound to a bis-phosphorylated segment of the transmembrane activator PZR/MPZL1 (PDB: 9MQ5). *Bottom middle:* open/active conformation of SHP2 modeled via AlphaFold2, with the tandem SH2 domains in the same configuration seen in PDB: 9MQ5. *Bottom right:* open/active conformation derived from SHP2^E76K^ (PDB: 6CRF), with the N-SH2 domain closer to the front of the PTP domain than in the AlphaFold2 model but the C-SH2 domain in the same general position and orientation. (**C**) SHP2 responds to inputs from a variety of signaling pathways, is activated by binding to phosphoproteins, and mediates a range of cellular outcomes through its catalytic and scaffolding functions.

*PTPN11* was first associated with human disease in 2001, when 9 mutations in this gene were linked to Noonan syndrome, a genetic disease marked by a range of developmental defects [24]. Many more pathogenic *PTPN11* mutations have been discovered since then. Interestingly, the majority of these mutations are missense (Figure 2A). By contrast, mutations that lead to a non-functional protein, such as frameshift mutations or premature stop codons, are virtually absent in humans. Indeed, the genotype *PTPN11*^−/−^, where no functional *PTPN11* copy is present, causes embryonic lethality [25–27]. At the same time, one wild-type copy of *PTPN11* (*PTPN11*^+/−^) is in principle sufficient for normal embryogenesis and cellular function. This is another reason why even heterozygosity for a *PTPN11* null allele is not typically present in patients with SHP2-associated disease (Figure 2B) [27]. However, in the background of impaired epidermal growth factor receptor (EGFR) signaling, co-inheritance of a *PTPN11* null allele severely exacerbated the observed phenotype in mice [28], suggesting that the cellular function in a *PTPN11*^+/−^ background is dependent on wider genetic context. In rare cases, when loss of heterozygosity occurs (*PTPN11*^+/−^ becomes *PTPN11*^−/−^) in somatic cartilage progenitor cells, cartilage tumors can form in a skeletal disorder called metachondromatosis (Figure 2B) [29,30]. In short, it is typically not the loss of *PTPN11* that causes disease; rather the presence of a mutated allele (*PTPN11*^+/m^) underlies SHP2-associated pathologies (Figure 2B). These mutant proteins operate through hypermorphic or neomorphic mechanisms and can cause both blood and solid cancers (Figure 2C,D), as well as germline developmental disorders known as ‘RASopathies,’ including Noonan syndrome (Figure 2B,D) [24,31,32]. 50% of the Noonan syndrome cases are caused by mutations in *PTPN11*, while the clinically distinct disorder Noonan syndrome with multiple lentigines (NSML) is almost exclusively *PTPN11*-related (Figure 2D). Both of these syndromes can also be caused by dysregulation of other components of the Ras/MAPK pathway, such as *NF1* or *SOS1*. However, the exact composition of patient symptoms and severity of clinical presentation is heavily dependent on the gene underlying the disorder. Similarly, the a large fraction of patients with juvenile myelomonocytic leukemia (JMML), a rare childhood blood cancer, harbor mutations in Ras/MAPK pathway components, including *PTPN11* (Figure 2D).

**Figure 2:**
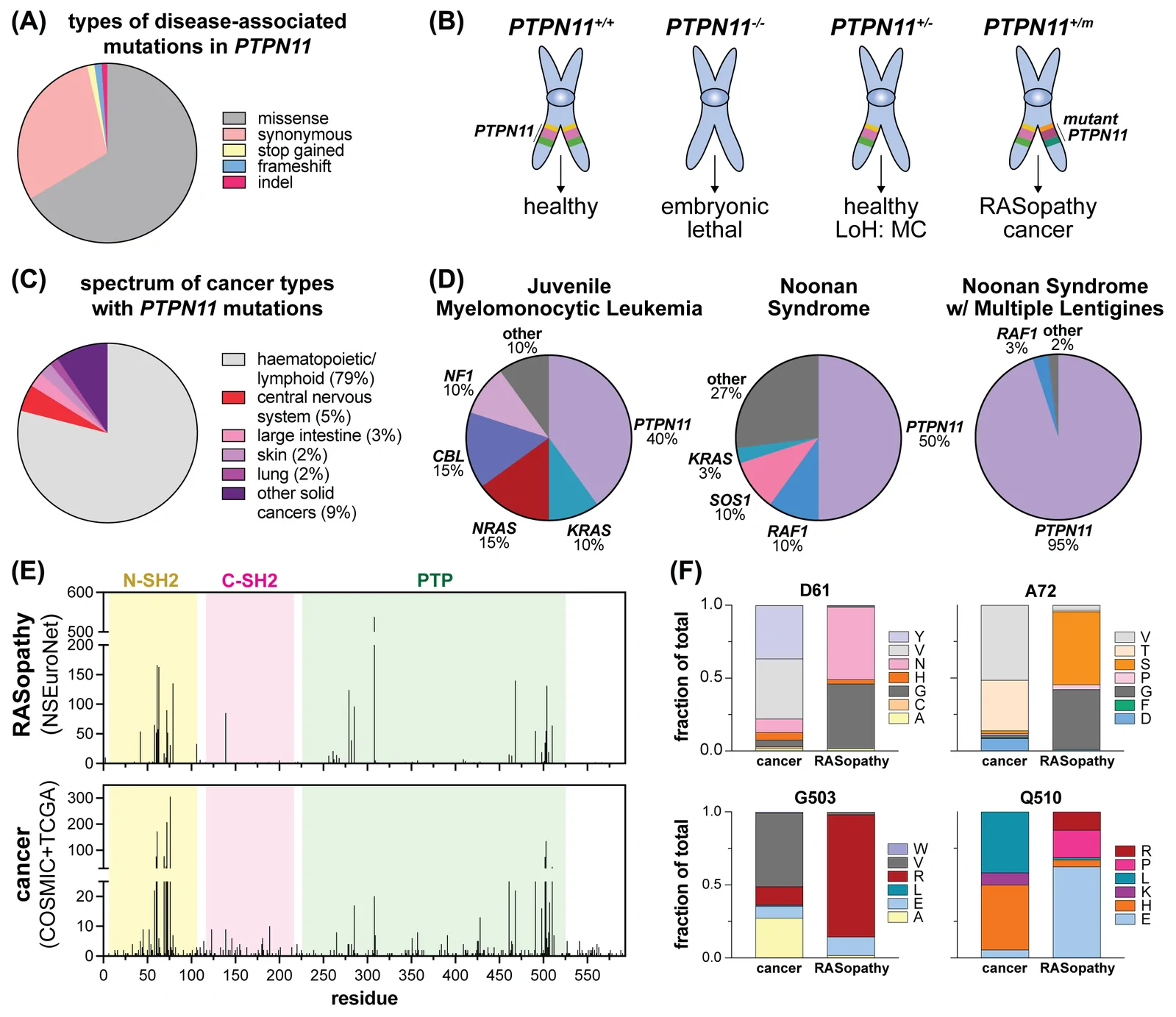
The disease landscape of *PTPN11*/SHP2 mutations (**A**) Genetic classification of mutations in *PTPN11* as provided in Ensembl. (**B**) Molecular genetics of *PTPN11*/SHP2. LoH = loss of heterozygosity; MC = metachondromatosis. (**C**) Distribution of *PTPN11* mutations across different cancer types. Data was derived from COSMIC, and low-frequency mutations (<5 entries) were excluded. (**D**) Pie charts for specific *PTPN11*-related diseases, showing disease-driving genes as identified in DNA sequencing of patient cohorts [24,33–38]. *NRAS, KRAS, NF1, SOS1*, and *RAF1* are components of the Ras/MAPK pathway. (**E**) Positions and frequencies of missense mutations (black bars) in SHP2 along its 593-residue sequence (Supplementary Tables S1 and S2). (**F**) Comparison of amino acid substitution frequencies for select SHP2 hotspot residues in cancer and RASopathy, highlighting disease-dependent mutational spectra. In panels (E,F), cancer mutation counts are derived from COSMIC and TCGA databases, and RASopathy mutation counts are derived from NSEuroNet.

To date, dozens of pathogenic missense mutations in SHP2 have been identified. They are scattered across all three globular domains but are rarely found in the disordered C-terminal tail. In both RASopathies and cancers, these mutations cluster into hotspots that largely map to key structural regions involved in auto-inhibition, catalysis, and protein–protein interactions (Figure 2E). However, nuances exist within these mutational hotspots: some residues in the same functional region are mutated more frequently than others, depending on the disease; and even at an individual position, the distribution of specific amino acid substitutions often differs between disease types (Figure 2F). The latter observation suggests that different mutations at the same site might perturb SHP2 structure and activity in different ways, with distinct signaling outcomes. Altogether, the large collection of known pathogenic mutations in SHP2 can be thought of as an extensive set of perturbations to the protein that alter the structural and signaling space of this enzyme in myriad ways. This makes SHP2 an ideal model system for exploring how mutations alter input–output relationships in a multi-domain protein. Finally, it is noteworthy that there are hundreds of variants of uncertain significance scattered across the whole length of SHP2, which represent largely unexplored sequence space that could yield new mechanistic insights [39].

In the present review, we discuss the mechanisms through which missense mutations in *PTPN11* perturb SHP2 structure and activity at the molecular level, and how this connects to changes in signal transduction at the cellular level. As SHP2 is a signaling protein with catalytic activity, we first focus on the mechanisms through which catalytic efficiency is altered by mutations. However, the non-catalytic roles of SHP2 are also critical to its signaling functions, and many pathogenic mutations have been identified that alter these catalysis-independent functions of SHP2. Thus, in the second part of the present review, we focus on insights into SHP2 function that emerged from investigating mutations that impact its non-catalytic properties. Finally, we discuss open questions related to SHP2 mutations and suggest future directions for the field.

## Many roads to altered SHP2 catalytic activity

Both gain-of-activity and loss-of-activity SHP2 mutations can lead to disorders with similar clinical presentation. This apparent inconsistency between catalytic activity changes and disease phenotypes has historically been puzzling [40,41]. Therefore, early biochemical studies on SHP2 mutations focused on elucidating their effects on enzymatic activity. These investigations were increasingly coupled with biophysical studies, including structural characterization of the auto-inhibited state of SHP2 and multiple active conformations, revealing how mutations simultaneously alter SHP2 catalytic activity and global domain arrangement [9–13,42–44]. More recently, high-throughput characterization of SHP2 mutations by cell-based deep mutational scanning (DMS) and by high-throughput microfluidic enzyme kinetics assays have provided a new level of depth in SHP2 structure–function analyses [45,46].

In the DMS datasets, signal primarily correlates with basal phosphatase activity [45]. Sites of high mutational sensitivity, whether activating or deactivating, tend to be highly conserved across SHP2 metazoan orthologs, consistent with these residues being important for SHP2 function (Figure 3A). Interestingly, despite similarly high conservation in both the N-SH2 and C-SH2 domains, very few mutations in the C-SH2 impact catalytic activity (Figure 3A). This suggests that, while the C-SH2 domain is important for SHP2 function, it is not a direct mediator of catalytic activity, unlike the N-SH2 or PTP domains. Notably, mutations in the C-SH2 domain are rare in both cancers and RASopathies (Figure 2E), hinting at catalytic activity as an important driver of pathogenicity. Many of the strongest mutational effects in the DMS datasets are at residues bearing prevalent cancer and RASopathy mutations (Figure 3B). While the high-frequency cancer mutations are all very activating, the DMS datasets show that high-frequency RASopathy mutations can be both activating and deactivating, suggesting different constraints on function in different disease contexts (Figure 3B) [45]. Thus, although catalytic activity may be important in some disease contexts, it is not the sole determinant of pathogenicity for SHP2. The diverse structural and biochemical mechanisms by which SHP2 mutations alter catalytic activity are discussed in detail in the subsequent sections.

**Figure 3:**
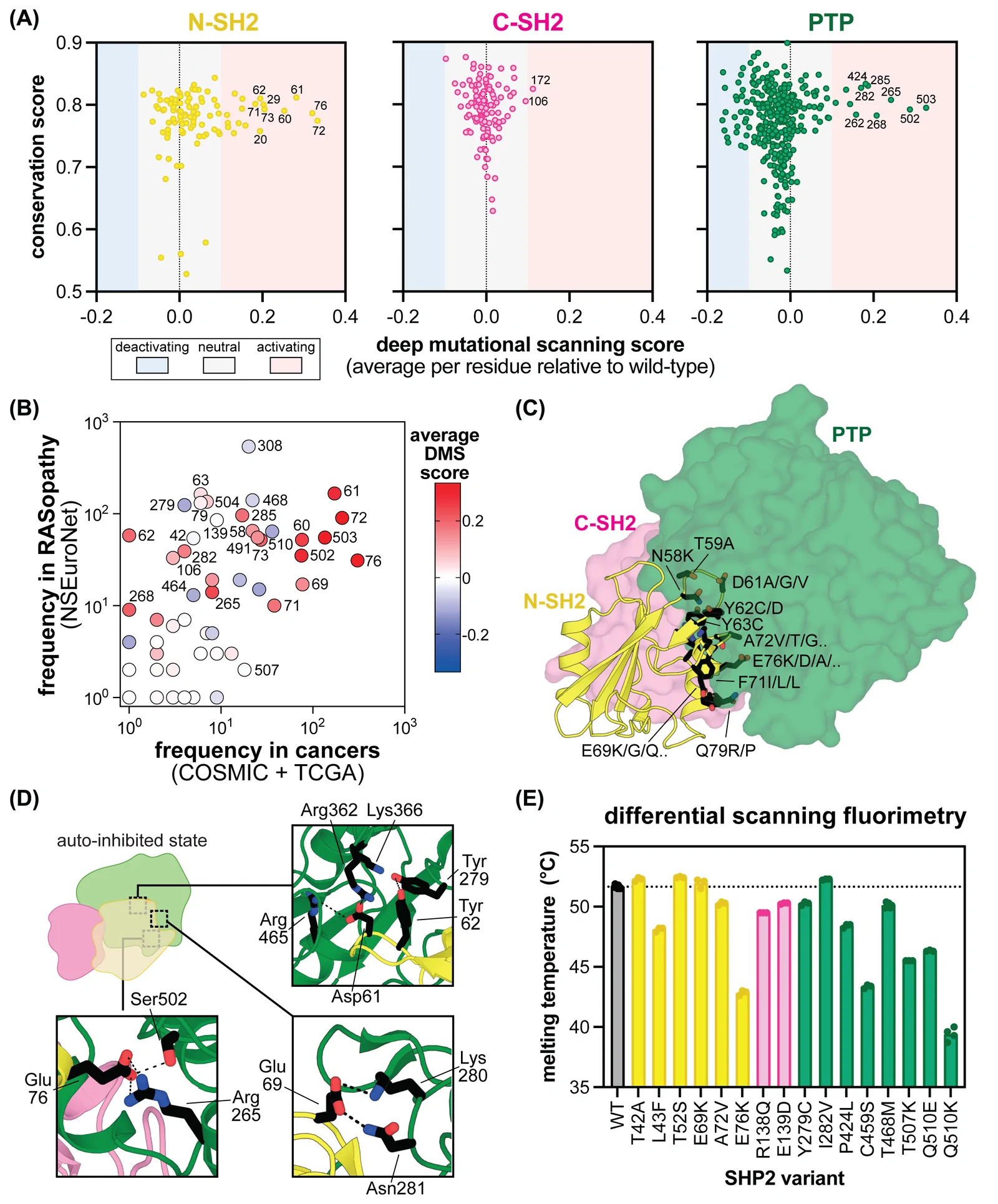
Distribution and conservation of high-frequency SHP2 mutation sites (**A**) Conservation score (Jensen–Shannon divergence [47], calculated from sequence alignment of 137 metazoan SHP2 orthologs) versus DMS score [45] (calculated as the average enrichment score per residue), showing stronger mutational effects in the N-SH2 and PTP domains than the C-SH2 domain, despite high C-SH2 conservation. Conservation and DMS scores can be found in Supplementary Table S2. The sequence alignment is provided as Supplementary File S1 with additional details in Supplementary Table S3. (**B**) Frequency of mutations at each SHP2 site in cancers (COSMIC + TCGA) versus RASopathies (NSEuroNet), colored based on average score from DMS. Only residues with at least one occurrence in both the cancer and RASopathy databases are shown. (**C**) Frequently mutated residues that make up the interface between the N-SH2 and PTP domains in the auto-inhibited state (PDB: 4DGP). (**D**) Interaction hotspots between the N-SH2 and the PTP domains (indicated by dashed circles) and Glu69 interactions with the PTP domain. (**E**) Melting temperatures for several SHP2 mutants discussed throughout the present review, measured via differential scanning fluorimetry (source data for melting temperatures: wild-type, E76K [45,48,49]; T42A, L43F, T52S [48]; R138Q, E139D [45,48]; A72V, E69K, Y279C, I282V, P424L, C459S, T468M [45]; T507K [45,49]: Q510K, Q510E [49])

### Auto-inhibitory interface mutations on the N-SH2 domain

As discussed above, the auto-inhibited state of SHP2 is stabilized by a large interface between the N-SH2 domain and the PTP domain. Interactions at this interface position the N-SH2 domain to occlude the catalytic center from phosphorylated substrates. The most common disease mutations on the N-SH2 side of this interface are found in both cancer and RASopathies and include G60A, D61G, E69K, A72V, T73I, and E76K/A (Figure 3C) [28,50,51]. Mutations on Tyr62, such as Y62D, also disrupt auto-inhibition but are rare in cancer patients (Figure 3B). These interface residues cluster into two major hotspots [52]: G60, D61 and Y62 on the N-SH2 domain are positioned close to the substrate-binding pocket on the PTP domain (Figure 3C,D). The negatively charged Asp61 sits in a positively charged center, surrounded by Arg362, Lys366 and Arg465, while Tyr62 engages Tyr279 via hydrogen bonding and π stacking (Figure 3D) [53,54]. At the same time, Ala72, Thr73 and Glu76 reside in a pocket between several loops in the PTP domain, with Glu76 directly interacting with Arg265 and Ser502 (Figure 3D). Strongly activating mutations that disrupt these auto-inhibitory interactions such as D61V, Y62D or E76K, are frequently identified in both RASopathy and/or cancer patients (Figure 3A).

There are nuanced differences in the molecular mechanisms by which mutations at the auto-inhibitory interface activate SHP2, with important therapeutic implications. Mutations that repel the PTP domain through increased steric hindrance or electrostatic repulsion, like D61Y and E76K, respectively, so strongly destabilize the auto-inhibited state that they usually confer resistance to SHP2 allosteric inhibitors, which are designed to trap wild-type SHP2 in its auto-inhibited state [10,55]. By contrast, merely removing key side chain interactions, as in D61G and E76A, more mildly disrupts auto-inhibition, and these mutants can still be inhibited by allosteric inhibitors [52,56]. While all of the aforementioned mutations exert their effects by disrupting the auto-inhibited state, it has been proposed that the E76K mutation also dysregulates SHP2 by Lys76 engaging in favorable interactions with negatively charged residues on the PTP domain in the active state [52]. However, these interactions have not been observed in experiments. Perhaps the most puzzling N-SH2 mutation on the N-SH2/PTP interface is leukemia-associated E69K. Unlike other interface residues, Glu69 is positioned outside of the two interaction hotspots and is in the vicinity of Lys280 and Asn281 [9,55] (Figure 3D). Although SHP2^E69K^ has comparable catalytic activity to other hyperactivating mutations on the N-SH2 domain, its melting temperature, as measured by differential scanning fluorimetry, is similar to wild-type SHP2, whereas most mutations that destabilized the auto-inhibited state have lower melting temperatures (Figure 3E) [45,57]. Moreover, SHP2^E69K^ is the only characterized auto-inhibitory interface mutant known to be inhibited by the allosteric inhibitor SHP099 [45,55]. This suggests that alternative modes of activation might be relevant for this mutant, beyond destabilization of the canonical auto-inhibitory interface.

### Destabilizing mutations in the hydrophobic core of the N-SH2 domain

The N-SH2 domain of SHP2 also harbors disease-associated mutations that are not directly at the auto-inhibitory interface but instead in the hydrophobic core of the N-SH2 domain. Our DMS study showed that most disease-associated N-SH2 core mutations, and nearly all other substitutions in the N-SH2 core, are activating (Figure 4A,B) [45]. These mutations dysregulate SHP2 by destabilizing the N-SH2 domain, reducing auto-inhibition as a consequence. Similar mutations in other SH2-domain-containing proteins, such as BTK and SH2D1A, have been found in human diseases and reduce the half-life of these proteins, consistent with a domain destabilization model [58–62]. For SHP2, biochemical analyses and cell signaling assays suggest that there is a functional trade-off that constrains which mutations are present in human diseases: while severely N-SH2-destabilizing mutations release auto-inhibition and thus have high phosphatase activity, they also ablate the phosphoprotein binding capability of the N-SH2 domain, thereby curbing signaling interactions [45]. By contrast, mildly N-SH2-destabilizing mutations, like L43F, modestly enhance SHP2 phosphatase activity without compromising phosphoprotein binding or downstream signaling (Figures 3E and 4B). This may explain why the highest-activity N-SH2 core mutations are not observed in RASopathies or cancers (Figure 4B). The functional constraints on N-SH2 destabilization as a mechanism for dysregulation reinforce the idea that SHP2 pathogenicity is not a function of catalytic activity but rather is a cooperative outcome of catalysis and protein–protein interactions. Finally, it is worth noting that aside from alleviating auto-inhibition, mutations in the N-SH2 domain can also alter SHP2 function by changing its ligand binding affinity and specificity, which will be discussed in later sections.

**Figure 4:**
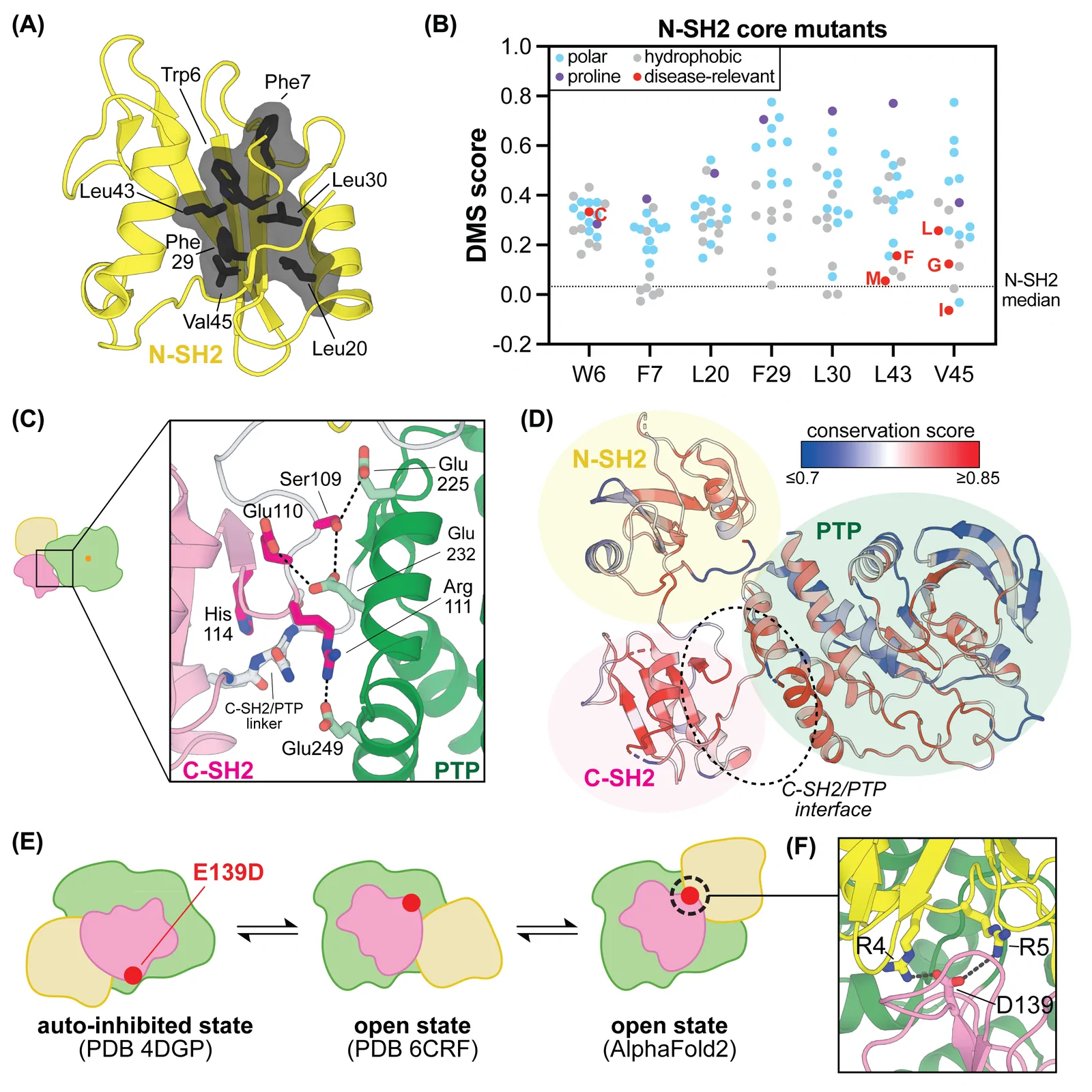
SH2 mutations outside of the N-SH2/PTP interface that increase SHP2 activity (**A**) Residues that make up the N-SH2 domain core for which an increase in activity was observed in DMS (PDB: 6ROY). (**B**) Enrichment scores from DMS [45] under high selective pressure (SHP2_FL_ co-expressed with v-Src_FL_) for each possible mutation at residues in the N-SH2 domain core. The median enrichment score of the entire N-SH2 domain is indicated as a dotted line. Mutations found in disease databases (ClinVar, TCGA, or COSMIC) are indicated in red and labeled with the one-letter code of the substituted amino acid. (**C**) Interactions between residues in the C-SH2 domain and PTP domain that stabilize the crystallographic open conformation of SHP2 (PDB: 6CRF, shown), as well as the AlphaFold2 open conformation (not shown). (**D**) Rendering of open conformation SHP2 (PDB: 6CRF), with conservation scores for each amino acid indicated in color, highlighting very high conservation at the C-SH2/PTP interface (dashed ellipse). (**E**) Location of E139D in different SHP2 conformational states. (**F**) Ion pairing between Asp139, Arg4, and Arg5 in molecular dynamics simulations of SHP2^E139D^ starting from the AlphaFold2 open conformation.

### C-SH2 interface mutations that impact SHP2 active conformations

Compared with the well-studied auto-inhibitory interactions between the N-SH2 and PTP domains, interdomain interactions that stabilize the SHP2 open conformations are less understood. The first indication of such interactions came from the open conformation crystal structure of SHP2^E76K^, which showed that C-SH2 residues Ser109, Glu110, Arg111, and Thr219 form contacts with a series of PTP domain residues (Figure 4C) [10]. Residues in this interface are highly conserved, suggesting a critical function (Figure 4D). Later, it was demonstrated experimentally that an ion pair between Arg111 on the C-SH2 domain and Glu249 on the PTP domain stabilizes the open conformation seen in the SHP2^E76K^ crystal structure [63], and this ion pair is also present in the alternate open conformation model via AlphaFold2 (Figure 1B) [45]. DMS, coupled with molecular dynamics simulations of these open conformations, revealed that the C-SH2/PTP interface is further stabilized by C-SH2 residue His114 docking into a pocket on the C-SH2/PTP linker (Figure 4C) [45]. These observations indicate that, beyond its role in mediating phosphoprotein interactions, the C-SH2 domain likely has the critical function of stabilizing SHP2 active conformations [63]. Notably, there is an extensive C-SH2/PTP interface in both the auto-inhibited state and the various active conformations, but this interface is not the same, as the C-SH2 domain rotates relative to the PTP domain during activation. A handful of disease mutations reside in the cleft between the C-SH2 and the PTP domains, such as E110K/A and T218A/S. These mutations are mildly activating via DMS and high-throughput enzyme kinetics assays [45,46], and they likely facilitate the reorientation of the C-SH2 domain during activation.

The C-SH2 domain also forms an interface with the N-SH2 domain that changes from the auto-inhibited to active state(s). However, unlike the C-SH2/PTP interface, which is conserved between the SHP2^E76K^ open conformation crystal structure and the alternate AlphaFold2 active state model, the C-SH2/N-SH2 interface differs between those two open states. This has implications for SHP2 dysregulation. For example, the E139D mutation in the C-SH2 domain, found in both Noonan syndrome and JMML patients, is hyperactivating, but this effect cannot be explained by its roles in the auto-inhibited state or the SHP2^E76K^ open state crystal structure. However, Glu139 is at the N-SH2/C-SH2 interface in the AlphaFold2-modeled active state (Figures 1B and 4E). Molecular dynamics simulations and mutant cycle analyses revealed that the E139D mutation selectively stabilizes this AlphaFold2-modeled active state by enhancing ion pairs with Arg4 and Arg5 on the N-SH2 domain (Figure 4F) [13]. This conformational biasing was further supported by NMR spectroscopy [64]. By biasing the active-state ensemble, E139D not only activates SHP2, but it also impacts its protein interaction specificity, which we discuss later.

### WPD loop mutations that enhance catalysis and auto-inhibition

Tyrosine phosphatases have several catalytic loops that collectively facilitate substrate dephosphorylation (Figure 5A). The WPD loop, named after the conserved residues Trp–Pro–Asp, is the most mobile of these loops. It moves between a “WPD-closed” and a “WPD-open” conformation that is critical for the full catalytic cycle (Figure 5B), but the closed state is specifically required for substrate dephosphorylation, as this is when Asp425 of the WPD motif is close enough to the catalytic center to act as a general acid and base. The structural determinants of WPD loop dynamics and mutational effects that alter these dynamics have been extensively explored in related tyrosine phosphatases, most notably PTP1B [65,66], and the learned principles can be applied to SHP2. For example, Pro180 in PTP1B (Pro424 in SHP2) stabilizes the WPD-open conformation by packing with Trp179 (Trp423) and Pro185 (Pro429), while restraining the main chain conformation of the loop (Figure 5B). The P180A mutation in PTP1B is activating and favors closure of the WPD loop [65]. Likewise, in deep mutational scans of both full-length SHP2 and its isolated PTP domain, most Pro424 mutations, including the pathogenic P424L mutation, increased catalytic activity (Figure 5C) [45]. For SHP2, however, WPD loop mutations have consequences beyond the isolated PTP domain. While the WPD loop does not directly participate in the auto-inhibitory interface, structural studies demonstrate that the WPD-closed conformation is incompatible with the auto-inhibited state, as it would clash with the N-SH2 domain (Figure 5A,D) [9,12]. As a result, mutations in and around the WPD loop that favor the WPD-closed conformation can also disrupt auto-inhibition by displacing the N-SH2 domain. Consistent with this, melting temperature measurements showed that the P424L mutation destabilizes the auto-inhibited state in a similar manner to N-SH2/PTP interface mutations (Figure 3E) [45].

**Figure 5:**
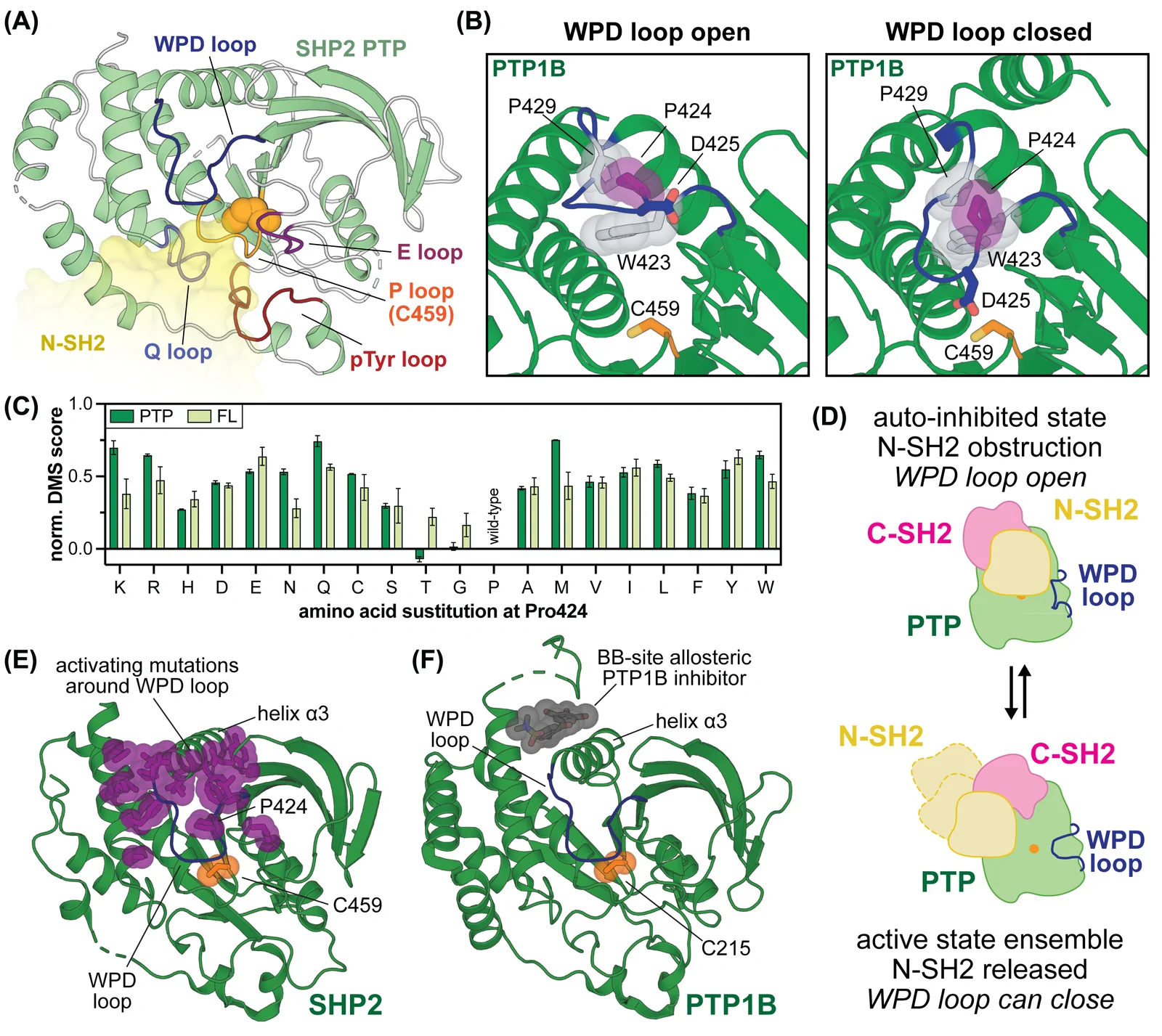
SHP2 activation via perturbation of WPD loop dynamics (**A**) Location of the key catalytic loops in SHP2. The N-SH2 domain is artificially superimposed onto the PTP domain to (i) indicate steric obstruction of the N-SH2 domain with the WPD loop closed conformation in the auto-inhibited state and (ii) highlight that residues on specific catalytic loops of the PTP domain are involved in maintaining auto-inhibition (PDB: 2SHP). (**B**) Renderings of the open and closed states of the WPD loop (blue) in PTP1B, but with key conserved residues labeled in SHP2 numbering (PDB: 5K9V, open, and 5K9W, closed). Images highlight the packing of Pro424 (purple) with Trp423 and Pro429 (gray). The distance between Asp425 (blue sticks) and the catalytic cysteine 459 (orange) changes as a function of loop movement and determines the catalytic activity of the PTP domain. (**C**) Mutational effects at Pro424 in full-length SHP2 and the isolated PTP domain, as measured by DMS [45]. SHP2_FL_ + c-Src_KD_ and SHP2_PTP_ + v-Src_FL_ datasets were each normalized using maximum absolute scaling. (**D**) The WPD closed conformation clashes with the auto-inhibitory N-SH2 domain in the SHP2 closed conformation; however, in the open state, the WPD loop can close. (**E**) Locations of activating mutations in proximity to the WPD loop in the SHP2 PTP domain, as seen by DMS (PDB: 3ZM0). (**F**) Binding site of the allosteric “BB site” inhibitor in PTP1B (PDB: 1T48).

Interestingly, DMS also revealed that mutations closer to the base of the WPD loop can increase PTP domain activity (Figure 5E) [45]. These mutations cluster around nearby helix ⍺3, which has been shown in PTP1B and SHP2, as well as related phosphatases SHP1 and YopH, to modulate WPD loop dynamics through an extended allosteric network [67]. This region also harbors a binding site for PTP1B allosteric inhibitors, known as the “BB site” (Figure 5F) [67]. The conservation of this allosteric network across several PTPs [68], coupled with the clear mutational sensitivity in SHP2 around this region, supports the idea that this pocket could be exploited for the development of novel allosteric modulators of SHP2.

### Active site mutations in the PTP domain that disrupt both auto-inhibition and catalysis

Unlike the WPD loop, the other catalytic loops of SHP2 are more directly involved in the auto-inhibitory interface (Figure 5A). As discussed above, many N-SH2 interface residues function by inserting into pockets formed by these catalytic loops (Figure 3D). Mutations on the N-SH2 domain side of this interface merely disrupt the N-SH2/PTP interface. By contrast, mutations on the PTP domain side have the potential to simultaneously disrupt auto-inhibition (Figure 3E) and alter the intrinsic enzymatic activity of the PTP domain (Figure 6A). The balance between increased exposure of the catalytic center and decreased catalytic efficiency results in mixed net-functional effects for PTP domain mutations at the N-SH2/PTP interface (Figure 6B,C). Some net-deactivating disease-associated mutations were characterized in early biochemical studies, and recent DMS and high-throughput enzyme kinetics have captured the full spectrum of this compensatory effect (Figure 6C) [44–46,69].

**Figure 6:**
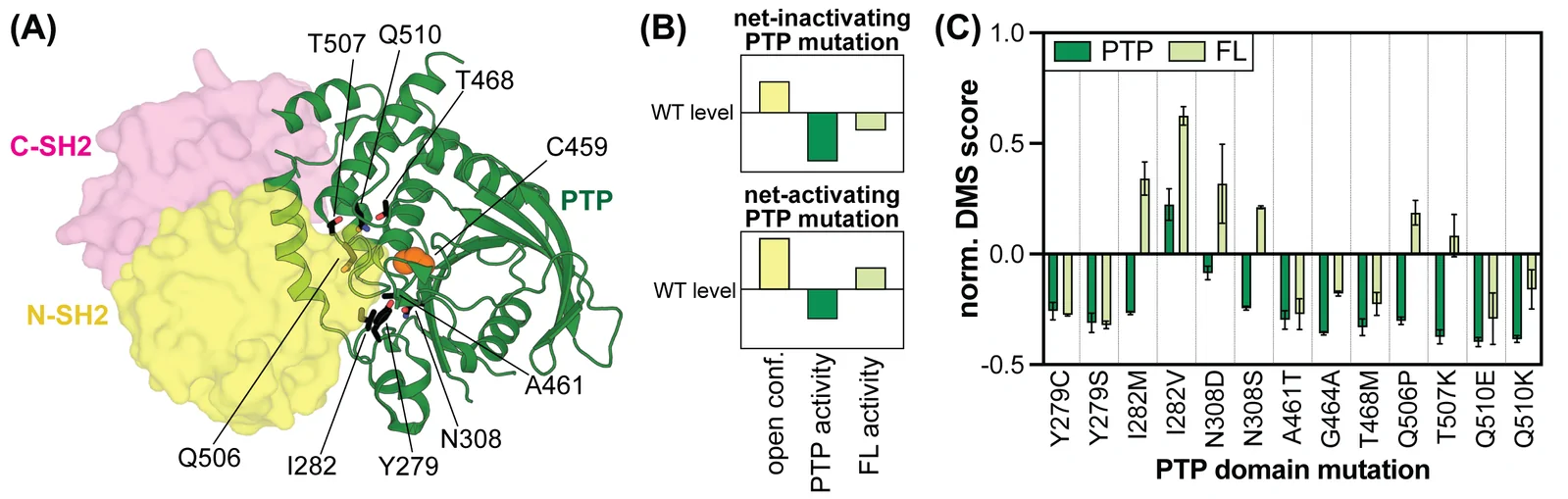
Compensatory effects of PTP mutations at the N-SH2/PTP interface (**A**) Locations of several disease-associated mutations on the SHP2 PTP domain, mapped onto the auto-inhibited structure (PDB: 4DGP). (**B**) Examples of outcomes for PTP mutations at the N-SH2/PTP interface. The level of compensation for disruption of intrinsic catalytic activity of the PTP domain (dark green bar) by disrupting auto-inhibition and favoring the open conformation (yellow bar) can lead to different effects on overall SHP2 activity (light green bar). (**C**) PTP domain mutational effects in full-length SHP2 and the isolated PTP domain, as measured by DMS [45]. SHP2_FL_ + c-Src_KD_ and SHP2_PTP_ + v-Src_FL_ datasets were each normalized using maximum absolute scaling.

The Y279C mutation, which is on the pTyr loop, has been shown to impair PTP engagement with the N-SH2 domain by abolishing interactions with Asp61 and Tyr62 (Figure 3D) [44]. However, Tyr279 is also critical for substrate docking, leading to an enzyme with reduced auto-inhibition but little-to-no catalytic activity (Figure 6C) [40,70]. Similarly, the net-activating disease mutation I282M decreases the catalytic activity while simultaneously alleviating auto-inhibition, whereas I282V maintains phosphatase activity, likely due to a more conservative change in side chain structure [45]. N308D and N308S, which are among the most common mutations in Noonan syndrome, are not immediately at the interface but rather in the second shell (Figure 6A). They appear to slightly decrease PTP activity in isolation, while activating full-length SHP2 (Figure 6C) [45,53,71]. The Q-loop is responsible for positioning a water molecule for the hydrolysis of the dephosphorylation intermediate, and mutations on its residues Gln506, Thr507, and Gln510 have been shown to inhibit catalysis yet promote SHP2 opening [44,49,69]. Notably, Q506P and N308D have recently been shown to alter the catalytic activity of the PTP domain via intra-domain allostery, shifting the WPD-loop conformational equilibria [71]. Some of these mutations have been found to also alter substrate-specificity of the PTP domain, which will be discussed in later sections [49,69]. Finally, this same activity-conformation balance has also been observed for P loop region mutations A461T, G464A, and T468M, which destabilize auto-inhibition but have a net-negative effect on catalytic activity [44].

All of the N-SH2/PTP interface-adjacent mutations described above favor the open conformation of SHP2, regardless of their net effect on activity. Moreover, they have the potential to increase SHP2 responsiveness in signaling pathways by facilitating protein–protein interactions, as will also be discussed in subsequent sections [44]. This is likely why NS and NSML patients have some overlap in developmental outcomes, despite some of their underlying mutations causing opposite effects on SHP2 catalytic activity [40]. Meanwhile, of the mutations that can reduce SHP2 enzymatic activity, only those that also alleviate auto-inhibition are frequently seen in diseases, whereas disease-relevant mutations that solely decrease PTP activity are extremely uncommon [45]. Historically, efforts to understand SHP2 pathogenicity have often looked at catalytic activity as a direct read-out for functional changes. However, comprehensive studies of SHP2 mutations, as summarized above, clearly demonstrate that catalysis alone does not dictate SHP2 function. As a signaling protein, SHP2 participates in signaling pathways through binding to upstream ligands, dephosphorylating downstream substrates, and mediating protein–protein interactions as a scaffold. Apart from SHP2 activity, ligand affinity and substrate specificity are also highly sensitive to mutations. In the next section, we discuss how SHP2 mutations impact its interaction with upstream and downstream signaling partners to alter signal transduction.

## Modulating the non-catalytic functions of SHP2 through sequence changes

Recent high-throughput explorations of SHP2 mutational effects [45,46], as well as focused, mechanistic studies that preceded it [50], have identified a number of disease-associated mutations that do not alter basal catalytic activity. The existence of these mutations hints at the possibility that SHP2 signaling could be dysregulated by perturbing its protein–protein interactions, which may or may not be relevant to its phosphatase activity. Indeed, SHP2 is known to function as a scaffolding protein in a variety of contexts [22,23,72,73]. The importance of these non-catalytic functions is illustrated by experiments showing that a catalytic-dead mutant of SHP2 (SHP2^C459S^) can provide partial rescue of cellular functions in an SHP2 knockout background [74–76]. Additionally, the SH2 domains of SHP2 can engage with phosphorylated tyrosine residues and protect them from dephosphorylation, which can sustain cellular signaling [77,78]. Thus, catalytically neutral, disease-associated mutations likely perturb the critical but non-enzymatic functions of SHP2, such as binding and scaffolding. Furthermore, mutations that do alter basal catalytic activity also have the potential to impact protein–protein interactions. In this next section, we examine how mutations affect protein–protein interactions of SHP2 through distinct mechanisms.

### Mutations that alter accessibility of interaction interfaces

One mechanism through which mutations can perturb protein–protein interactions is by altering the accessibility of interaction surfaces (Figure 7A). As discussed previously, many pathogenic SHP2 mutations, whether they enhance or decrease catalytic activity, shift its conformational ensemble toward an open state. In addition to exposing the catalytic site, these mutations can also put the SH2 domains in a more binding-competent state or expose other binding interfaces, with clear effects on downstream signaling. For example, many common NSML mutations, such as the aforementioned Y279C/S, are on the PTP domain, at the N-SH2/PTP interface, where they lower catalytic activity but also disrupt auto-inhibition (Figure 6). These mutations cause SHP2 to favor SH2-mediated interactions and prolonged engagement with binding partners, in turn, leading to increased Ras/MAPK signaling and a cellular phenotype comparable to that of hyperactive SHP2 variants [42,44]. Notably, this enhancement in ligand binding, caused by a shift in the conformational ensemble, is a common outcome of all mutations that disrupt the auto-inhibitory interface, whether or not they disrupt the catalytic capacity of the PTP domain. This leads to the well-observed convergence of disease phenotypes between pathogenic mutations of diverse catalytic capabilities.

**Figure 7:**
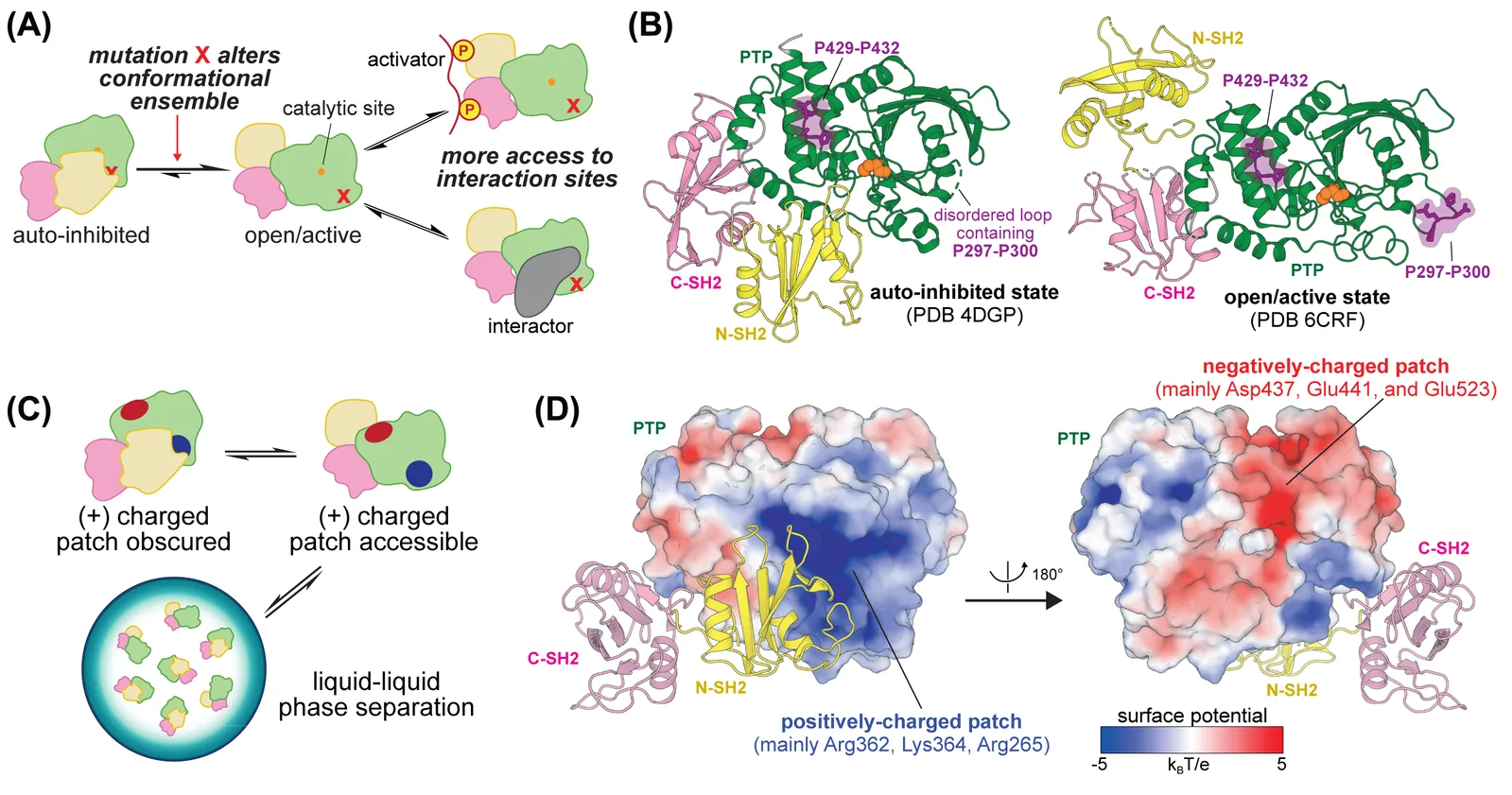
Altered access to protein interaction surfaces caused by disease-associated mutations (**A**) Mutations that alter the conformational ensemble of SHP2 can alter access to binding sites: the SH2 domains can become more individually binding-competent and better oriented to bind bis-phosphorylated proteins, the PTP active site is accessible, and other interaction surfaces may also be more accessible. (**B**) Position of the PxxP motifs on the PTP domain (purple) in the SHP2 closed (PDB: 4DGP) and open conformation (PDB: 6CRF). Both PxxP motifs have the same degree of solvent accessibility in both conformational states. (**C**) A positively-charged patch on the PTP domain is increasingly exposed in the open conformation of SHP2, allowing for intermolecular interactions that mediate liquid–liquid phase separation (LLPS). (**D**) Electrostatic surface potential of the PTP domain in the auto-inhibited state of SHP2 (PDB: 4DGP), highlighting obstruction of the positively charged patch as well as the location of the negatively-charged patch.

There are other binding sites on SHP2, outside of the PTP active site and the canonical phosphoprotein binding pockets on the SH2 domains. For example, a recent study revealed that the PTP domain of SHP2 has a binding site for the DH domain of the Ras guanine nucleotide exchange factor SOS1 [79]. However, this binding site is obscured by the N-SH2 domain in the auto-inhibited state. Mutations that shift the conformational equilibrium of SHP2 toward an open state have enhanced the ability to recruit SOS1 to the plasma membrane, where it can activate Ras and drive MAPK activation in a manner that is completely independent of SHP2 catalytic activity [79]. These findings further challenge the prevailing view that SHP2 regulates Ras/MAPK signaling exclusively through its catalytic activity. Instead, this model suggests that SHP2 is an evolutionarily conserved scaffold that promotes SOS1 recruitment and subsequent Ras/MAPK pathway activation. This interaction provides a potential explanation for the pathogenicity of mutations on residues in the SHP2–SOS1 interface, such as Q-loop mutants, Y279C, and I282V [79].

Additionally, the surface of the PTP domain has two PxxP motifs (Pro297–Pro300 and Pro429–Pro432; Figures 1A and 7B). Studies have shown that the SH3 domain of the tyrosine kinase c-Src can bind these PxxP motifs, leading to c-Src activation [80–82]. As with the SOS1 example, SHP2 catalytic activity is expendable for c-Src activation via this mechanism, and NSML mutants that sample the SHP2 open conformation more frequently show increased interaction with c-Src [81,82]. It has been suggested that this could be due to altered accessibility of the PxxP motifs in the open conformation of SHP2. However, these motifs are solvent-exposed to a similar extent in the auto-inhibited and the open conformations of SHP2 (Figure 7B) [9,10]. Moreover, the C459S mutant, which is strongly biased toward the open state (Figure 3E) [12,74,83], was previously shown to activate c-Src to the same extent as wild-type SHP2, suggesting that alternative or additional mechanisms likely underlie the biased activation of c-Src by NSML mutants [80]. One plausible explanation is that the Pro429–Pro432 PxxP motif, which lies on the WPD loop, is more conformationally constrained in the auto-inhibited state because it is pushed into a WPD out conformation by the N-SH2 domain (Figure 5D), and this may impact its binding compatibility with the c-Src SH3 domain; however, this has not been directly tested.

Finally, several studies have reported that specific SHP2 mutants, such as SHP2^E76K^, induce LLPS at rates above wild-type SHP2, causing increased downstream signaling (Figure 7C) [84,85]. These types of biomolecular condensates are generally driven by proteins that can engage in specific multivalent interactions [86]. Indeed, in a study focused on receptor tyrosine kinase signaling, SHP2 was shown to form condensates with several phosphorylated kinases, most notably FGFR2 [87]. This occurred through multivalent interactions of the SH2 domains of SHP2 with the phosphorylated cytoplasmic tail of FGFR2. Interestingly, for SHP2, phase separation also appears to be driven by intermolecular electrostatic interactions involving the PTP domain: a positively charged patch, mainly composed of residues Arg362 and Lys364, with contributions from Arg265, interacts with a negatively charged patch made up of Asp437, Glu441, and Glu523 (Figure 7C,D) [84]. A similar finding was recently reported for the SHP2 paralog SHP1 [88]. Notably, the cationic patch on the SHP2 PTP domain is masked in the auto-inhibited state, which is why a variety of mutants that prefer an open conformation were shown to increase LLPS relative to wild-type SHP2, irrespective of their catalytic activities (Figure 7C,D). Of note, in the FGFR2-SHP2 LLPS study, the catalytic-dead SHP2^C459S^ mutant was used to prevent rapid dephosphorylation of FGFR2, which would remove the binding sites that underlie the condensate formation [87]. However, as this mutant is also biased toward the open conformation [12,74,83], this might have contributed to the observed condensation effect. Together, these findings highlight an important principle: pathogenic mutations in multi-domain enzymes often act by reshaping conformational ensembles, and if individual states in these ensembles are related by large interdomain rearrangements, mutations may unveil binding surfaces and significantly impact protein–protein interactions or the assembly of higher order species like biomolecular condensates.

### Mutations that alter the intrinsic affinity or specificity of protein–protein interactions

Each globular domain of SHP2 has a well-defined ligand binding pocket that can engage binding partners in a selective manner. Whereas the previous section addressed mutations that alter access to these binding pockets or other binding interfaces, many disease-associated mutations occur within these pockets. These binding-site mutations have the potential to alter the intrinsic binding affinity or specificity of an individual domain, which can impact protein–protein interactions and downstream signaling in unique ways. Select examples of such mutations are discussed here.

The T42A mutation in the SHP2 N-SH2 domain is a common mutation in Noonan syndrome [89], and substitutions at the analogous position have been identified in other SH2-containing proteins, highlighting the functional relevance of this residue in SH2 domains more generally [90–92]. Thr42 in SHP2 is outside of the auto-inhibitory interface, and mutations at this site are not substantially activating [50,48,64,89,93]. However, Thr42 lies in the pTyr binding pocket of the N-SH2 domain, where it directly engages the pTyr residue on the ligand via a hydrogen bond (Figure 8A). Remarkably, the T42A mutation, in which this hydrogen bond donor is lost, has been shown to increase affinity for phosphopeptides and phosphoproteins [48,89,93]. This causes an increase in activated, ligand-bound SHP2, albeit through a different mechanism than mutations that disrupt auto-inhibition such as SHP2^E76K^ (Figure 8B). Molecular dynamics simulations have suggested that this is due to a re-orienting of the pTyr residue upon loss of this key hydrogen-bond, resulting in a more favorable binding pose [48]. However, this re-orientation was not observed in recent crystal structures of N-SH2^T42A^ [64]. Notably, the change in binding affinity upon the T42A mutation appears to be sequence-dependent: not all phosphopeptides show the same enhancement in binding affinity [48]. While the precise molecular mechanism for this change in specificity is not known, it likely involves mutation-dependent reorientation of key residues lining the ligand-binding pocket (e.g. Lys55) or a mutation-dependent change in binding mode for select ligands [48]. The altered binding affinity and specificity caused by the T42A mutation has important functional consequences in the cell, as proximity-labeling proteomics has shown that this mutation strongly remodels the SHP2 interactome [94]. One notable specific outcome is that SHP2^T42A^ displays an increased interaction with the transmembrane protein PZR/MPZL1 [48,94]. A similar enhancement in the SHP2-PZR interaction is also observed in NSML and is an important mediator of pathogenic signaling [81,82]. However, NSML mutants have enhanced PZR binding due to their predisposition for the open conformation, whereas the Noonan syndrome T42A mutation converges on this same outcome through an enhancement in intrinsic affinity of the N-SH2 domain for a phosphosite on PZR (Figure 8B).

**Figure 8:**
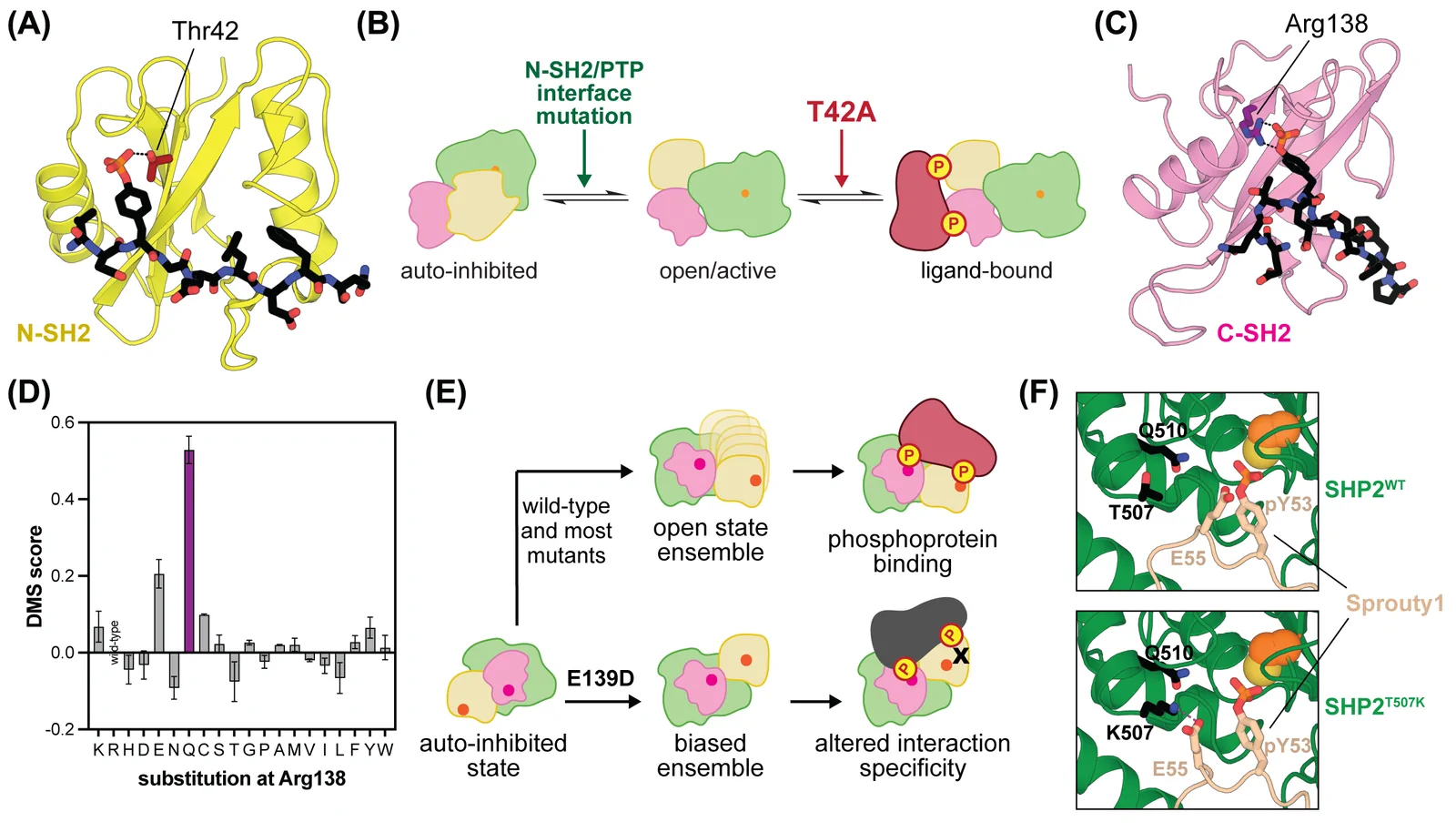
Mutation-altered binding affinity and specificity of the SHP2 globular domains (**A**) Structure of the N-SH2 domain bound to a phosphopeptide, highlighting hydrogen bonding of Thr42 to the phosphoryl group on the ligand (PDB: 6ROY). (**B**) Coupled equilibria of SHP2 conformational change and phosphoprotein binding. Canonical N-SH2/PTP interface mutations alter the initial conformational equilibrium, whereas the T42A mutation alters the second binding equilibrium, both leading to enhanced occupancy of SHP2-phosphoprotein complexes. (**C**) Structure of the C-SH2 domain bound to a phosphopeptide, highlighting ion pairing between Arg138 and the phosphoryl group on the ligand (PDB: 6R5G). (**D**) Enrichment scores from DMS under high selective pressure (SHP2_FL_ co-expressed with v-Src_FL_) for each possible mutation at Arg138 in the C-SH2 domain [45]. (**E**) The E139D mutation stabilizes a specific open conformation of SHP2 that could impact its interaction specificity with bis-phosphorylated proteins. (**F**) AlphaFold2 model suggests how the T507K mutation alters the substrate specificity of SHP2 by remodeling active site electrostatics to enhance dephosphorylation of Sprouty1 at pY53.

As discussed above, the SHP2 C-SH2 domain has a lower mutational burden than the N-SH2 domain, but it is still functionally important, both for stabilizing the active state and for mediating protein–protein interactions. A particularly intriguing disease-associated mutation in the C-SH2 domain is R138Q, a somatic cancer mutation [32]. Arg138 is the key arginine in the conserved FLVR motif of SH2 domains, and it is critical for coordinating phosphotyrosine in the ligand binding pocket (Figure 8C) [95,96]. The R138Q mutation has been shown to abolish binding of the C-SH2 domain to peptide ligands [48,97]. This mutant is additionally unable to support a response to EGFR stimulation in cells, likely due to the loss of scaffolding and localization capabilities of the C-SH2 domain [49,97]. However, SHP2^R138Q^ showed more interactions when compared with SHP2^WT^ in a proximity-labeling study, suggesting that this mutant can acquire new interactions, perhaps through mislocalization [94]. Intriguingly, in our DMS experiment, the Arg-to-Gln mutation was the only substitution at Arg138 that substantially increased phosphatase activity (Figure 8D), and it mildly lowers the melting temperature of SHP2 (Figure 3E), suggesting that there might be more to learn about this mutation [45]. It is noteworthy that the N-SH2 counterpart to Arg138, Arg32, does not have any reported disease-associated mutations, underscoring differences in how the two SH2 domains contribute to SHP2 regulation and signaling through phosphoprotein binding. Notably, several pathogenic mutations affecting the analogous arginine in other human SH2 domains have been reported [98]. In PIK3R1, the R649W mutation shows reduced interaction between p85α and IRS-1, impaired signaling, causing SHORT syndrome [99–101]. In Zap70, R37G similarly disrupts cellular signaling pathways [102]. It is worth noting that, like SHP2, PIK3R1 and Zap70 are also tandem SH2-containing proteins. Dual SH2 domains frequently function as a cooperative binding unit that requires structural coordination while also being asymmetric in terms of affinity and specificity.

Previously, we described how the E139D mutation activates SHP2 by stabilizing a specific open state (Figure 4E,F). This mutation does not significantly alter the ligand binding affinity or specificity of the isolated C-SH2 domain [48,89]. However, we recently found that this mutation diminishes binding to some but not all bis-phosphorylated SHP2 binding partners in co-immunoprecipitation experiments [13]. Consistent with this co-immunoprecipitation phosphoproteomics experiments with SHP2 constructs bearing just the tandem SH2 domains also showed that the E139D mutation alters the interactome of SHP2 [97]. One plausible explanation for this effect is that, by constraining the positioning of the N-SH2 and C-SH2 domains relative to one another, the E139D mutation may predispose SHP2 to bind some bis-phosphorylated ligands better than others, with different preferences than wild-type SHP2 (Figure 8E).

Like the SH2 domains, mutations in the PTP domain can also alter substrate binding preferences. Specifically, two mutations in the Q loop (Figure 5A) that alter catalytic efficiency have also been shown to alter substrate specificity. This was first observed for the T507K mutation, where the introduction of a positive charge in the substrate-binding pocket confers a preference to a corresponding negative charge on the substrate (Figure 8F) [69]. Similarly, Q510K also introduces a positive charge in the same region of the substrate-binding pocket, with a similar change in substrate specificity, albeit with a more dramatic decrease in catalytic efficiency and stronger overall destabilization of the auto-inhibited state [49]. Finally, we note that mutations outside of canonical binding pockets on SHP2 can also potentially remodel binding interfaces. For example, SHP2 has a proline-rich region on its tail (residues 559–568, which are notably absent in SHP1), which could potentially bind to SH3 domain-containing proteins. The L560F mutation sits in the middle of this region and has the potential to perturb SH3 binding, or even change what SH3 domain-containing protein binds to SHP2, although studies have yet to demonstrate this.

## Future directions

The SHP2 field is currently in the privileged position where we have gathered structural, biochemical, cellular, and patient-level information on many SHP2 variants. By layering complementary methods, we are able to trace how a subtle structural perturbation propagates through signaling networks to yield a specific phenotype. Such an integrated framework offers a model for dissecting disease-associated variants with complex, nuanced effects. In this outlook, we first discuss some pressing open questions related to SHP2 variants, then we discuss additional methods and tools that would augment the interrogation of SHP2 mutational effects.

### Underexplored aspects of SHP2 mutational effects

#### Mutations with elusive structural mechanisms of dysregulation

Our ability to explain the structural basis for mutational effects in SHP2 requires that we know the functionally relevant conformations that SHP2 can adopt. Until recently, we exclusively had structures of the auto-inhibited state of SHP2, which only clarified how mutations at the auto-inhibitory interface exert their effects [9]. Elucidation of the structures of an increasing number of active conformational states and ligand-bound states of SHP2 is providing new insights into some of the more mysterious mutational effects [10,11,13,43,64,82]. This is best exemplified by the E139D mutation, which stabilizes a conformational state that was first revealed by AlphaFold2 modeling and is distinct from the only published crystal structure of active SHP2 at the time [13]. However, there remain several other disease-associated mutations observed via DMS to be activating, for which existing structural models provide no obvious explanation [45]. For example, D106A on the N-SH2/C-SH2 linker is a documented pathogenic mutation that is activating, but Asp106 does not participate in clearly meaningful interactions in any reported SHP2 structure. Several lines of evidence indicate that SHP2 adopts an ensemble of open conformations [11,13,82], and it is likely that alternate conformations remain to be seen to explain some of the more mysterious mutational effects observed via mutational scanning. Thus, there is a continued need for structural studies on SHP2 mutants.

#### Mutations that alter the redox sensitivity of SHP2

Many PTPs, including SHP2, are regulated by oxidation, primarily at the catalytic cysteine residue [103,104]. In SHP2, two other nearby cysteine residues (Cys333 and Cys367) are also redox active, and biochemical studies have suggested that these “backdoor” cysteines can form disulfide bonds with the catalytic Cys459 to stabilize it in a reversibly oxidized state [105]. These studies were corroborated by a crystal structure of the Noonan syndrome N308D mutant obtained from crystals grown under mildly oxidizing conditions, which revealed a disulfide bond between Cys367 and Cys459 (PDB: 6ATD) [106]. Further biochemical analysis demonstrated that the N308D mutant is more susceptible to oxidation than wild-type SHP2, both in the context of a near-full-length construct and the isolated PTP domain [106]. Thus, although full-length SHP2^N308D^ is more catalytically active than SHP2^WT^ due to disrupted auto-inhibition, in the presence of reactive oxygen species, this mutant may actually have less overall phosphatase activity while still remaining in a more open conformation. The precise molecular mechanism underlying the enhanced redox sensitivity of SHP2^N308D^ is not yet known, but these observations suggest that other disease mutants could be differentially susceptible to catalytic inactivation by oxidation.

#### Effect of mutations on SHP2 localization and compartment-specific function

SHP2 is present in the cytosol, nucleus, and mitochondria. While the first report of SHP2 presence in mitochondria dates back to 2005 [107], its role in mitochondrial biology remains poorly defined. Interestingly, clinical symptoms of patients with NS and NSML partially overlap with those of patients with mitochondrial disorders, suggesting a potential role for mitochondrial dysfunction in SHP2-driven pathology and justifying further research in this direction [108]. Wild-type SHP2 regulates the inflammasome through interaction with ANT1 and can also interact with components of the electron transport chain [109]. Further studies aimed at characterizing the protein–protein interactions of SHP2 in mitochondria are likely to reveal additional functions.

SHP2 mutations can alter mitochondrial function in distinct ways. Oncogenic SHP2 variants, such as SHP2^D61G^ and SHP2^E76K^, have been shown to impair mitochondrial function and promote oxidative stress in cells [85,110,111]. How much of this dysregulation stems from their hyperactivity versus enhanced scaffolding capability remains unclear. Additionally, in a proximity-labeling proteomics study, we recently found that several SHP2 variants exhibit increased mitochondrial localization compared with wild-type SHP2 and showed that some of these mutants further alter mitochondrial respiration [94]. Our results suggested that conformational state or protein stability may influence the mitochondrial import of SHP2, but the precise mechanisms underlying this phenomenon are not known. Finally, it is noteworthy that the presence of SHP2 in cells, whether wild-type or mutant, drives the expression of genes involved in oxidative phosphorylation and glycolysis, suggesting an additional regulatory role for SHP2 on mitochondrial function that could be independent of its localization in this organelle [49].

#### Mutational effects on the tumor-suppressive functions of SHP2

SHP2 rose to fame as the first PTP that, in contrast with many other tyrosine phosphatases, amplifies proliferative signaling and functions as a proto-oncoprotein. However, SHP2 has tumor-suppressive functions in specific tissues and cancer types, such as liver [112], cartilage [113], glioma [114], myeloma [115], and colon cancer [17,116]. The repression of SHP2 function, whether through genetic deletions, increased degradation, or inhibitory protein–protein interactions, can have an oncogenic effect in these contexts. Thus, SHP2 is a context-dependent regulator of important cellular processes, and the duality in its function in cancer remains to be fully decoded. The effects of inactivating missense mutations on the tumor-suppressive signaling functions of SHP2 remain unclear. However, all SHP2 variants associated with liver cancer showed loss-of-activity in our DMS study [45], making it plausible that in these cases, loss-of-activity causes loss of tumor-suppressive ability. Future studies will require examining the functional consequences of these loss-of-activity mutations in the appropriate cellular context or in animal models of the relevant cancer types.

#### Pathogenic mutations that impact SHP2 regulation by post-translational modifications

Beyond the activation of SHP2 by SH2 engagement of phosphoproteins, there is evidence for additional layers of SHP2 regulation, predominantly through phosphorylation [117]. There are 34 known phosphosites in SHP2 according to PhosphoSitePlus [118], with 13 sites in the disordered C-terminal tail. SHP2 is phosphorylated by protein kinase A (PKA) on two residues in the SH2 domains (Thr73 in the N-SH2 domain and Ser189 in the C-SH2 domain), which inhibit phosphatase activity [119]. Interestingly, DMS in yeast found that T73D/E substitutions, which have the same resulting charge as phosphorylation on this residue, are instead activating [45]. This likely reflects intrinsic SHP2 activity, whereas cell-based studies where PKA is present show reduced activity for the T73D mutant [119]. Moreover, mutations on Thr73 have been identified both in RASopathies and in cancer, with varying effects on catalytic activity (neutral or increased activity). For example, T73I has been shown to increase activity, likely due to steric hindrance at the auto-inhibitory interface by the resulting, bulky isoleucine [50,119]. By contrast, the pathogenic mechanism for T73S, which both retains the PKA recognition motif R-R-X- [S/T] and is catalytically neutral, remains unclear. On the other hand, phosphorylation of Ser189 by PKA inhibits SHP2 function [119], likely by forming electrostatic interactions with positively charged Arg5 on the N-terminus. Positively charged mutants S189K/R, in turn, appear activating in the mutational scanning results, perhaps because electrostatic repulsion with Arg5 disrupts the auto-inhibited state. SHP2 is also phosphorylated on two serines in the C-terminal tail (Ser576 and Ser591) by Protein Kinase C, but these phosphorylation events have not been shown to alter SHP2 catalytic activity [120]. Disease-associated mutations on Ser576 have been observed, but their mechanism of action has not been explored.

In addition to the described Ser/Thr phosphosites, three tyrosine-phosphorylation sites have received extensive attention: Tyr62, which is in the N-SH2 domain, and Tyr542 and Tyr580 in the disordered C-terminal tail. Tyr62 is hyperphosphorylated in receptor tyrosine kinase-driven cancers, suggesting an important signaling axis that requires further investigation [121–123]. Furthermore, Y62D is a recurrent Noonan syndrome mutation that is highly activating, which has led the field to speculate that phosphorylation of this site might be activating [124–126]. However, while the Y62D mutation definitively activates SHP2 by destabilizing the auto-inhibited state [126], the structural and signaling consequences of Y62 phosphorylation have not yet been fully elucidated. By contrast, the tail phosphotyrosine sites in SHP2 are well-studied. Upon activation of the platelet-derived growth factor receptor (PDGFR), SHP2 is phosphorylated on Tyr542, creating a binding site for the SH2 domain of the adaptor protein Grb2. Here, SHP2 functions as a scaffold to recruit Grb2/SOS1 to the plasma membrane in response to receptor tyrosine kinase activation [22,23]. Similar phosphorylation-dependent Grb2 recruitment has been described for Tyr580 in response to either PDGFR or EGFR activation [72]. In addition to mediating protein–protein interactions, biochemical experiments using stable phosphotyrosine mimetics at these tail sites have demonstrated that they can activate SHP2 through intramolecular interactions with the SH2 domains [15,16]. Moreover, mutation of Tyr542 and Tyr580 to Phe reduces the ability of SHP2 to activate the Erk pathway upon EGFR stimulation [127]. For both tail tyrosines, rare mutations to cysteine have been described in patients, but the pathogenic signaling consequences of these mutations are unexplored [39,128]. Finally, it should be noted that mutations near these sites could also modulate SHP2 phospho-regulation. For example, N544T, a rare cancer mutation [128], would destroy the obligate pYxN motif Grb2-binding motif [2], thereby preventing Grb2 recruitment to Tyr542. These observations underscore the need to carefully examine the functional effects of mutations at or near regulatory phosphosites in SHP2.

### New approaches and tools to study SHP2 sequence–structure–function relationships

#### Probing SHP2 conformational dynamics

As discussed above, the active state of SHP2 is likely an ensemble of multiple open conformations [13,14,129]. These conformations may include partially open states or allosterically stabilized states that contribute to SHP2 function and regulation. Biases between these states caused by mutations likely contribute to different levels of phosphatase activation, ligand accessibility and specificity, and susceptibility to inhibition by drug molecules. Experimentally observing these different states and their transitions would provide mechanistic details about SHP2 activation, inhibition, and signal rewiring by mutations. Biophysical methods including HDX-MS [44,126], NMR spectroscopy [12,64], and differential scanning fluorimetry [13,45,57,63] have been applied to SHP2 mutants to characterize their conformational states. On the other hand, more direct observation of conformational transitions using single-molecule fluorescence resonance energy transfer and single-molecule magnetic tweezers has recently been demonstrated [43,130]. Further development of these biophysical approaches will empower the field to identify specific conformational states and determine their relative lifetimes/stabilities. Critically, these experimental approaches could be coupled with computational methods to model the SHP2 conformational ensemble, including molecular dynamics with enhanced sampling, which have been applied to SHP2 previously [14,129–131], or various recently developed deep learning-based approaches [132–134], to gain an atomistic picture of the SHP2 conformational ensemble. Ultimately, measuring how this ensemble is perturbed by SHP2 mutations, substrates, ligands, inhibitors, and post-translational modifications will clarify the unexplained nuances of SHP2 (dys)regulation and lay a foundation for understanding the regulation of other multi-domain signaling proteins.

#### Deconvolution of mutational effects on SHP2 conformation, activity, and binding

As accumulating evidence suggests, the signaling and pathogenic functions of SHP2 are a combinatorial outcome of its catalytic activity and its protein–protein interactions. However, it remains challenging to decouple these two properties in SHP2 mutation studies. Signaling assays in a conformation-, activity-, or binding-restricted background could provide novel insights. For example, SH2 mutations that affect ligand specificity (e.g. T42A [48]) or PTP mutations that alter substrate specificity (e.g. T507K or Q510K [49,69]) could be examined in the context of the open conformation E76K mutation instead of a wild-type background. Alternatively, the signaling consequences of auto-inhibitory interface mutations could be studied on top of the catalytically dead C459D/E mutation so that the isolated mutational effects of conformational changes can be examined [79]. Mutations that disrupt N-SH2 or C-SH2 function, such as R32A and R138A [135], could also be combined with disease-associated mutations to examine how the SH2 binding pockets contribute to pathogenic signaling. This experimental design will unveil the contribution of each of the two aspects to the functional effects of SHP2 mutations.

#### Phosphoproteomics to assess mutant-driven dysregulation of cell signaling

Phosphoproteomics is a key approach for directly probing the state of signaling networks by providing a global snapshot of the cellular phosphorylation state while also enabling site-specific identification of modulated sites. Previous phosphoproteomics work on the pharmacological inhibition of SHP2 revealed six distinct temporal patterns in tyrosine phosphorylation dynamics, indicating nuanced roles for SHP2 at different stages of EGF signaling [136]. The study also identified novel potential substrates by showing increased phosphorylation upon SHP2 inhibition. Interestingly, a large class of sites exhibited decreased phosphorylation upon SHP2 allosteric inhibition, evidence that SH2 domain binding to phosphoproteins shields specific phosphotyrosine sites from dephosphorylation by other phosphatases. These results have expanded our understanding of the complex role of SHP2 in orchestrating signaling networks [136]. To date, phosphoproteomics has not been applied to dissect the specific signaling consequences of disease-associated SHP2 mutations, which can affect both catalytic and scaffolding functions of SHP2. Extending phosphoproteomic approaches to SHP2 mutants would reveal how individual mutations reshape downstream signaling, whether by altering enzymatic activity or by changing SH2 interactions. Phosphoproteomic profiling would be especially valuable for mutations that could rewire signaling pathways, such as the T42A mutation [48]. Moreover, integrating phosphoproteomic data with existing interactome and transcriptome datasets would enable a more comprehensive view of how individual SHP2 mutations reshape signaling networks [49,94].

#### Mutant-compatible SHP2 inhibitors

Given the central role of SHP2 in promoting proliferative signaling, it has been the focus of numerous drug discovery efforts. In receptor tyrosine kinase-driven cancers, where SHP2 is typically not mutated but is important for oncogenic signaling, inhibitors have been shown to suppress cancer cell proliferation [137]. The most promising inhibitors in this space are designed to allosterically stabilize the auto-inhibited state of the enzyme [138]. A direct consequence of this mechanism of action is that these allosteric inhibitors are not effective against some hyperactive variants, such as SHP2^E76K^, which rarely inhabit the auto-inhibited state [10,12]. Interestingly, recent high-throughput examination of SHP2 allosteric inhibition across many mutants revealed that, while the first-in-class inhibitor SHP099 binds to and stabilizes the auto-inhibited state of SHP2, next-generation inhibitors in this family, such as TNO155, RMC4630, and GDC1971, actually bind preferentially to an intermediate, partially open state [46]. Further biophysical and structural studies will undoubtedly provide a clearer picture of this mode of inhibition. Nonetheless, there remains a need to develop novel mutant-compatible SHP2 inhibitors for the treatment of RASopathies such as Noonan syndrome and cancers such as JMML.

As an alternative to allosteric inhibitors, orthosteric inhibitors, disruptors of protein–protein interactions, and degraders are promising avenues to inhibit pathogenic SHP2 mutants [139]. Owing to the conserved nature of PTP active sites and their highly charged catalytic pocket, selective orthosteric inhibitors of PTPs with good cell permeability have traditionally been difficult to design [140]. Nonetheless, some recent investigations have produced cell-active, orthosteric inhibitors that show varying degrees of selectivity for SHP2 over SHP1 (4- to 40-fold), demonstrating that this avenue may be feasible [141,142]. These active site inhibitors should, in theory, bind preferentially to active/open variants of SHP2, potentially complementing the allosteric inhibitors discussed above. Beyond the active site, there is also the potential to inhibit SHP2 function by blocking its SH2 domains, thereby disrupting its protein–protein interactions and localization. Indeed, peptide-based inhibitors of both the SHP2 N- and C-SH2 domains have recently been reported [143,144]. These peptides are derived from known SHP2 binding partners and display selectivity for SHP2 as a result. While cell permeability with these peptide-based inhibitors remains challenging, it is noteworthy that microinjection of an N-SH2-targeting peptide in zebrafish was able to rescue developmental defects driven by SHP2^D61G^, demonstrating the potential of these molecules to pharmacologically target pathogenic mutants [143].

Another promising strategy to disrupt mutant SHP2 signaling is targeted protein degradation. Most SHP2 proteolysis targeting chimeras (PROTACs) developed to date rely on allosteric inhibitors as starting scaffolds [145–148]. While these molecules show increased effectiveness in suppressing SHP2 signaling when compared with allosteric inhibition alone, they are still unable to target mutants that do not significantly inhabit the auto-inhibited state. In an innovative alternative approach, bivalent proteins bearing a SHP2-binding monobody fused to an E3 ligase (bioPROTACs) have also been developed [149]. These bioPROTACs were designed to bind to SHP2 surfaces that are accessible in multiple conformational states and thus are effective at degrading both SHP2^WT^ and SHP2^E76K^. Finally, there have been some nascent efforts to design mutant-specific SHP2 inhibitors as well. Researchers have targeted the NSML-associated Y279C mutation, exploiting the nucleophilicity of the acquired cysteine residue to develop a mutant-selective covalent inhibitor [150]. Thus far, this strategy is only a preclinical proof-of-concept, and extension to other mutations might be challenging. It is also unclear whether inhibition of the catalytic activity of NSML mutants would be most efficacious or if perturbation of protein–protein interactions is required to suppress pathogenic signaling. Nonetheless, the development of diverse modalities that can modulate the signaling functions of pathogenic SHP2 mutants holds promise for the treatment of SHP2-driven diseases.

## Concluding remarks

An emerging theme in the investigation of SHP2 variants is that changes to catalytic activity in SHP2 are not the sole driver of disease. Moreover, gain- or loss-of-activity does not correlate clearly with type of disease (e.g. RASopathies versus cancer). Instead, changes to the conformational space that SHP2 inhabits appear to drive a large share of pathogenicity through a variety of mechanisms, including increased activity and altered exposure of interaction surfaces. It is clear that categorizing SHP2 mutations is a nuanced and complex task [151]. SHP2 mutations that have different biochemical or structural effects on protein function can converge on the same cellular outcomes (Figure 9). Conversely, mutations that cause similar, direct perturbations to protein structure and dynamics can yield unique cellular phenotypes. This highlights the value of approaching each mutational study from multiple perspectives, to not only determine how the mutation perturbs SHP2 structure, but also how it impacts protein–protein interactions, protein stability, subcellular localization, modulation of parallel signaling pathways, and ultimately cell state. In principle, this holistic view of mutational effects could be applied to any multi-domain signaling enzyme.

**Figure 9:**
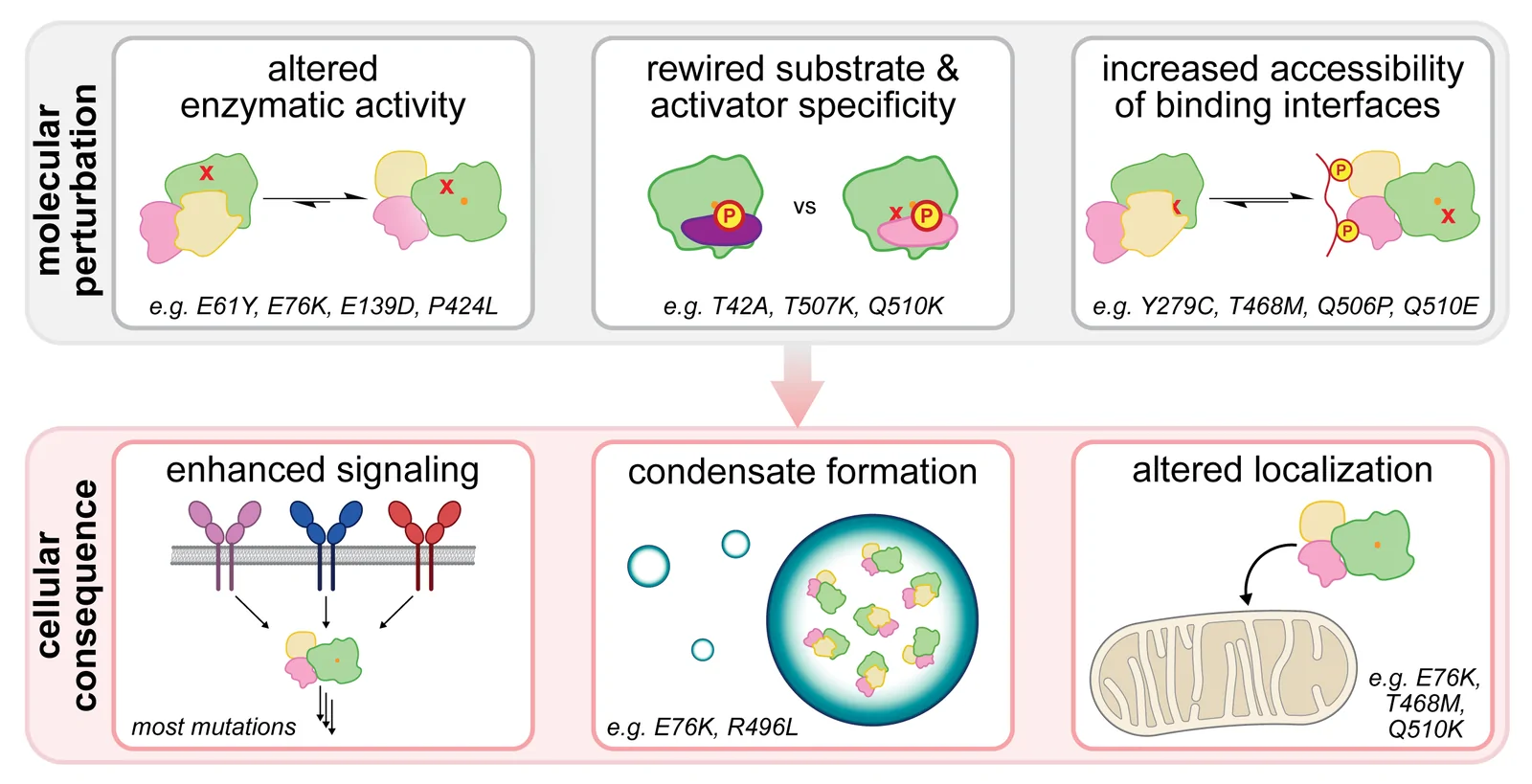
SHP2 dysregulation by a broad spectrum of mutational effects There are several different types of molecular perturbations to SHP2 structure and biochemical activity (*top*), which can alter SHP2 behavior in a cellular context in a variety of ways (*bottom*).

## Supplementary Material

Supplementary File

Supplementary Tables S1-S3

## Data Availability

Data sharing is not applicable to this paper.

## Footnotes

i **SH2:** Src Homology-2 domains. These domains bind phosphorylated tyrosine-residues and are frequently found in signaling enzymes as well as adaptor proteins. **SH3:** Src-Homology-3 domains. These domains recognize proline-rich regions. **PDZ:** this domain is an acronym for the first proteins in which it was observed: PSD-95, Dlg-1, and ZO-1. PDZ domains bind to the C-terminus of target proteins through beta sheet augmentation and facilitates interaction of receptor proteins to the cytoskeleton. **FERM:** another acronym-named domain (F for 4.1 protein, E for ezrin, R for radixin, and M for moesin). FERM domains are involved in localizing proteins to the plasma membrane.

ii **SHP2:** Src homology 2-domain containing protein tyrosine phosphatase 2.

## Open Access

Open access for this article was enabled by the participation of Columbia University in an all-inclusive Read & Publish agreement with Portland Press and the Biochemical Society.

## Abbreviations

C-SH2: C-terminal SH2
DMS: deep mutational scanning
JMML: juvenile myelomonocytic leukemia
LLPS: liquid–liquid phase separation
N-SH2: N-terminal SH2
NSML: Noonan syndrome with multiple lentigines
SH2: Src-homology 2
PDGFR: platelet-derived growth factor receptor
PKA: protein kinase A
PROTACs: proteolysis targeting chimeras
PTP: protein tyrosine phosphatase
